# Supplementary material for: Spatiotemporal dynamics of 53BP1 dimer recruitment to a DNA double strand break
Source: Nat Commun. 2020 Nov 13;11:5776. doi: 10.1038/s41467-020-19504-3 (PMC7666136; doi:10.1038/s41467-020-19504-3)
Supplement: Supplementary file 1 — Supplementary Information [file 41467_2020_19504_MOESM1_ESM.pdf]

Supplementary Information

Lou et al. “Spatiotemporal dynamics of 53BP1 dimer recruitment to a DNA double strand break”.

Supplementary Figures 1-7

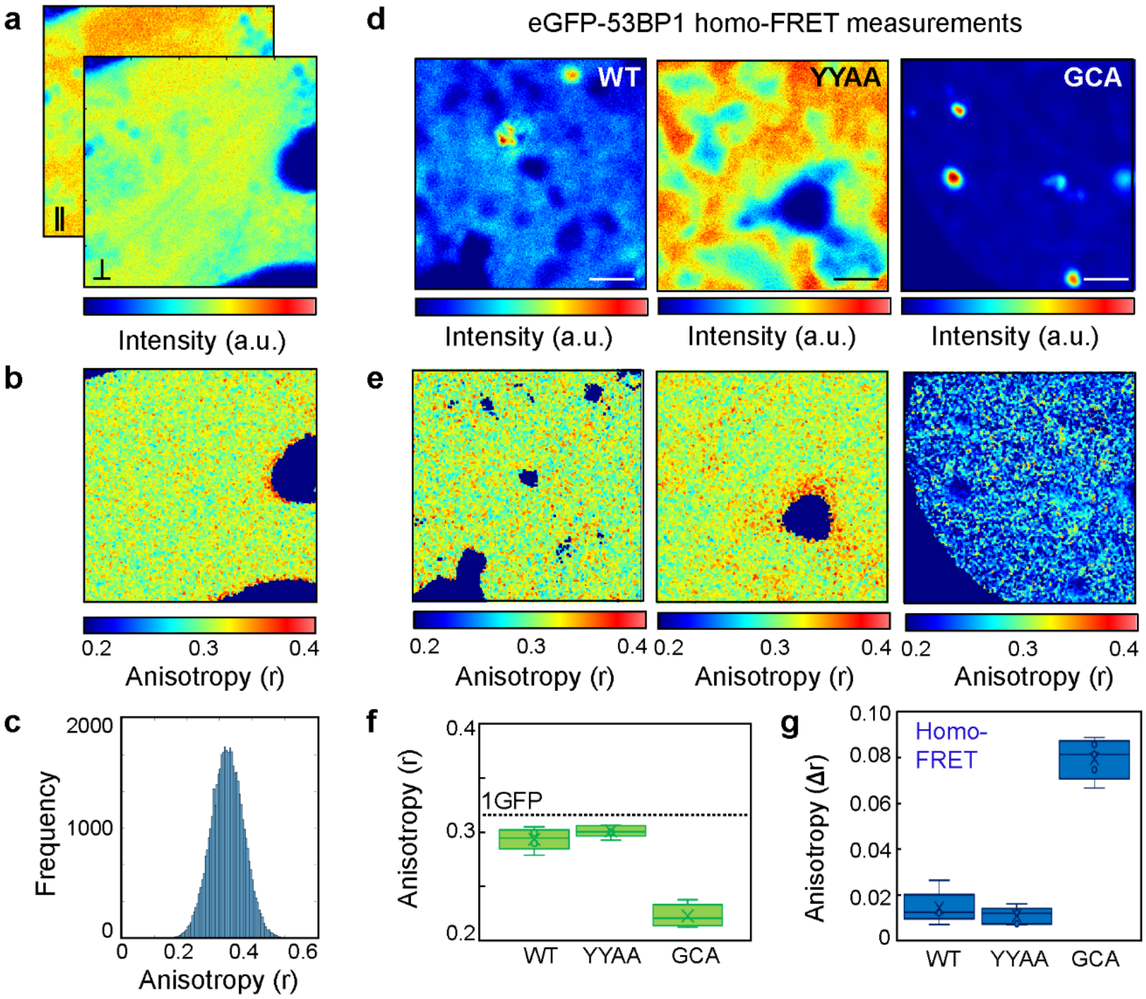

Supplementary Figure 1. Fluorescence anisotropy imaging microscopy (FAIM) of eGFP-53BP1 homo-FRET as an alternative readout of 53BP1 self-association. a. A FAIM acquisition of a live DIvA cell expressing free eGFP and the resulting fluorescence intensity images with polarisation parallel ($I_{\parallel}$) versus perpendicular ($I_{\perp}$) to excitation. **b.** Fluorescence anisotropy image of the eGFP acquisition presented in (a). **c.** Fluorescence anisotropy histogram of the eGFP anisotropy image presented in (b) (mean r value of 0.32 in the absence of homo-FRET)**. d-e.** Fluorescence intensity (d) and anisotropy (e) images of live DIvA cells expressing eGFP-53BP1 (WT), eGFP-53BP1^{YY1258,1259AA} (YYAA) and eGFP-GCA-53BP1 (GCA). **f.** Mean anisotropy r value of 53BP1 WT versus the 53BP1 mutants YYAA and GCA detects different degrees of depolarisation with respect to free eGFP when calculated across multiple cells (N = 6 cells, two biological replicates). **g.** Mean delta r (Δr) value of 53BP1 WT versus the 53BP1 mutants YYAA and GCA reports the degree of homo-FRET exhibited by these different constructs (N = 5 cells, two biological replicates). Box and whisker plots in (f)-(g) show the minimum, maximum, sample median, and first versus third quartiles. (Scale bars, 2 µm).

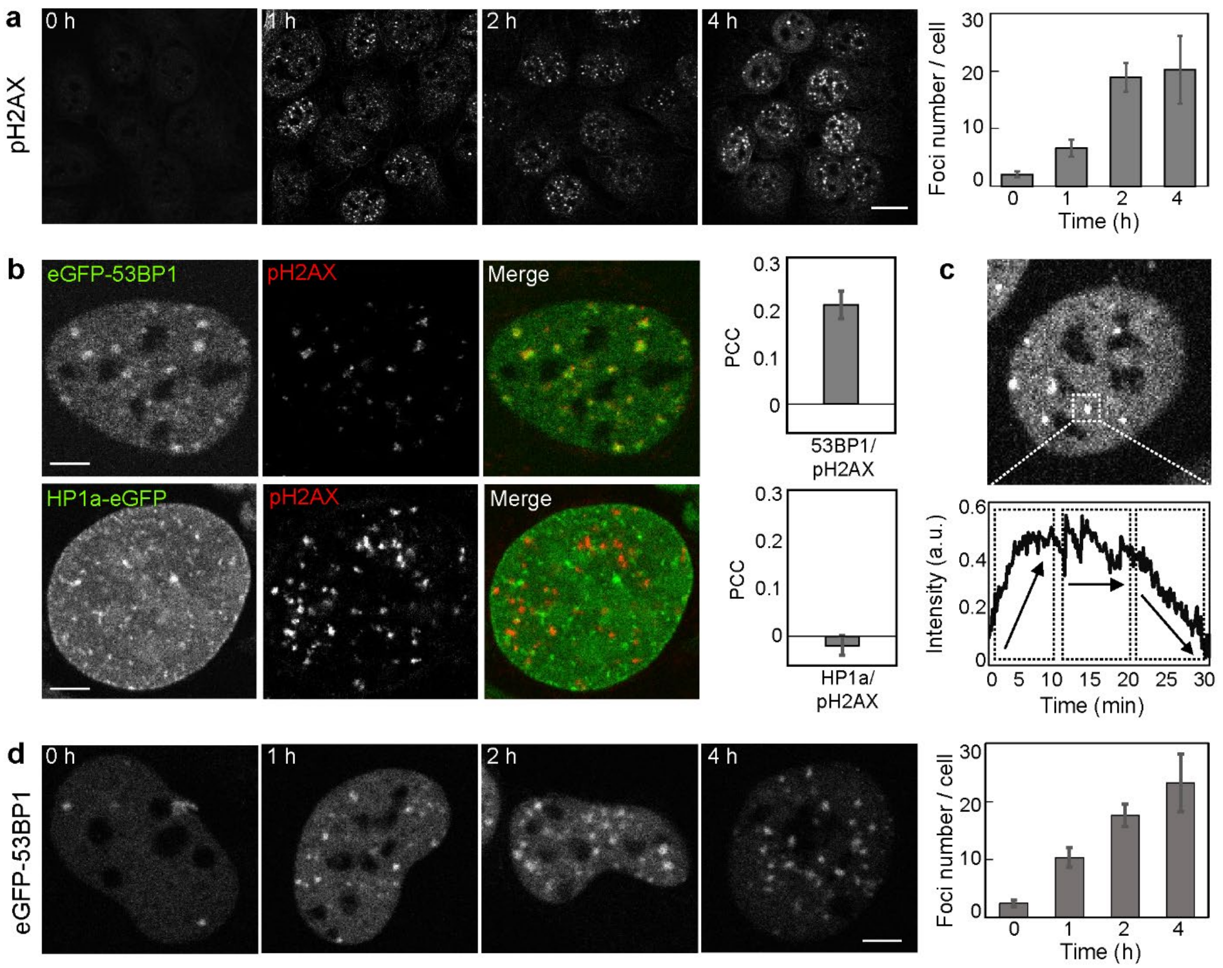

Supplementary Figure 2. Spatial and temporal kinetics of DSB foci formation in fixed versus live DIvA cells. a. Immunofluorescence against γH2AX in fixed DIvA cells reveals DSB foci formation to occur within 1 h of addition of 300 nM of 4OHT (Scale bar, 10 μm). **b.** Co-localisation of γH2AX immunofluorescence with eGFP-53BP1 (top row) versus HP1α-eGFP (bottom row) expression in fixed DIvA cells reveals: (1) eGFP-53BP1 to be an accurate read out of DSB foci localisation in live cell studies and (2) DIvA DSBs to be predominately localised in euchromatin (Scale bar, 5 μm). **c.** Expression of eGFP-53BP1 in live DIvA cells reveals foci formation to be dynamic and persist on average for 30 min. **d.** Expression of eGFP-53BP1 in live DIvA cells reveals DSB foci formation to occur within 1 h of addition of 300 nM of 4OHT. (Scale bar, 5 μm). Error bar indicate SEM.

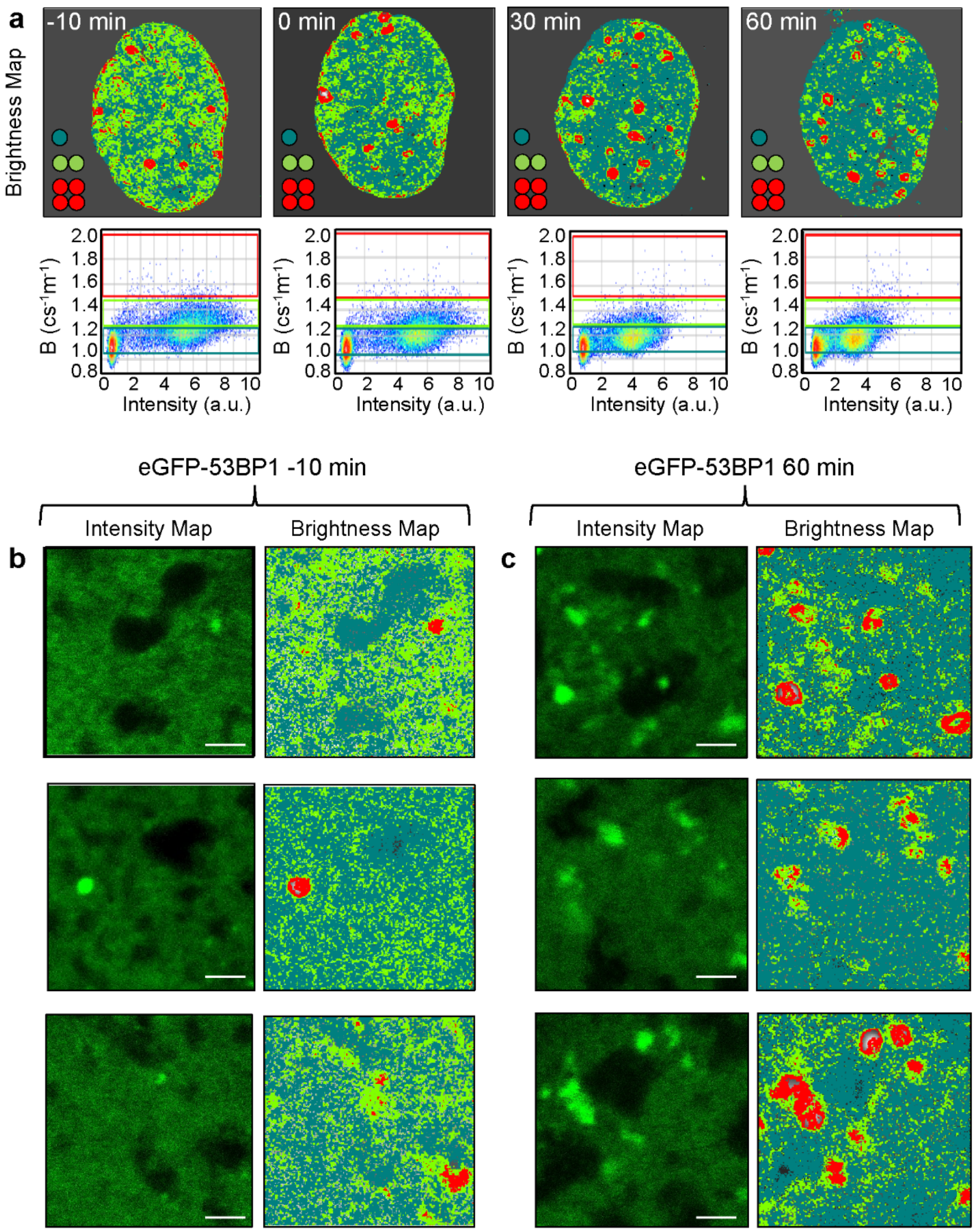

Supplementary Figure 3. Number and Brightness (NB) analysis of the spatiotemporal redistribution in eGFP-53BP1 oligomerisation upon DSB induction in live DIvA cells. a. Brightness maps of the confocal time series of eGFP-53BP1 foci formation presented in the main text figure Fig. 2c-d (top row) with corresponding intensity vs brightness scatterplots (bottom row). **b-c.** Representative NB FFS data acquisitions from regions of interest (ROIs) within different DIvA cell nuclei before (b) and 60 min after addition of 4OHT (c). (Scale bar, 2 μm).

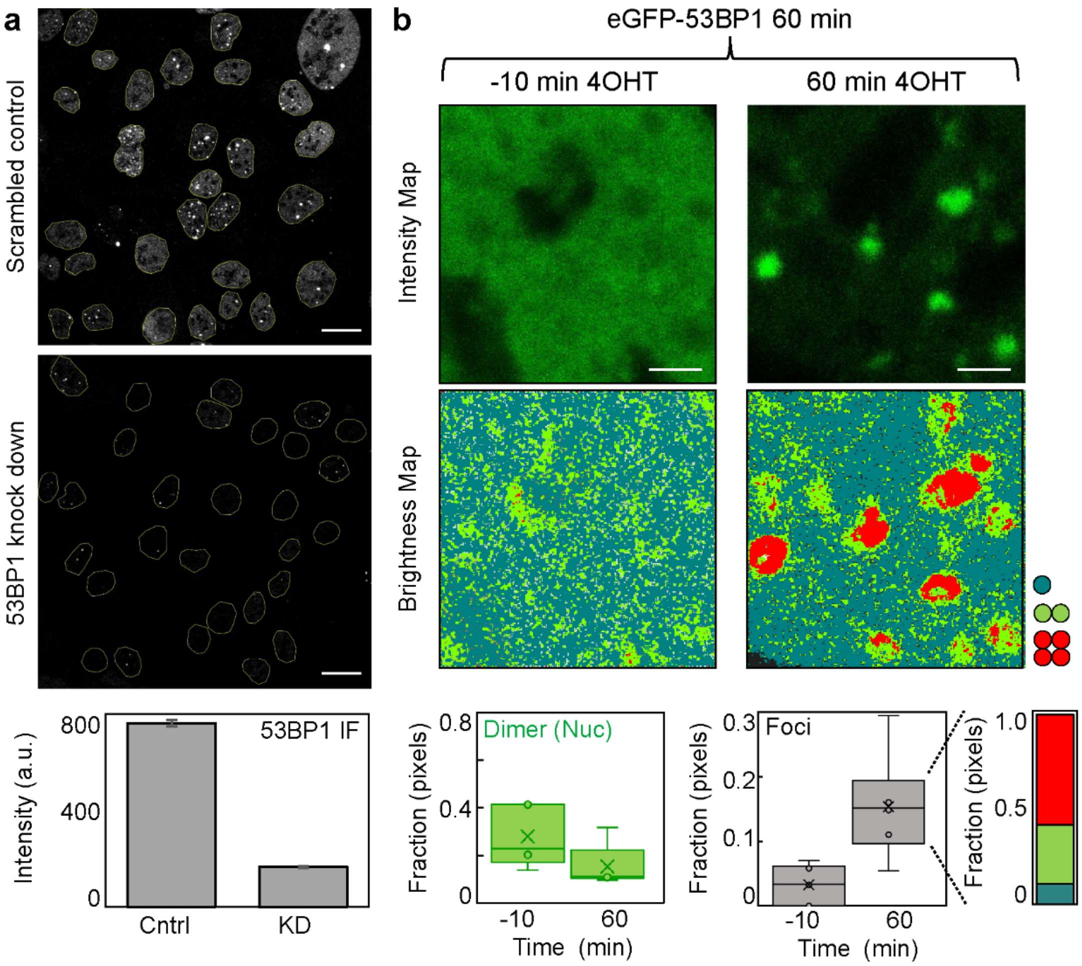

Supplementary Figure 4. eGFP-53BP1 expression versus endogenous 53BP1 in live DIvA cells and the impact this non-fluorescent pool of 53BP1 has on 53BP1 oligomerisation. a. Representative fluorescence intensity images of 53BP1 immunofluorescence in DIvA cells treated with scrambled siRNA (top panel) versus 53BP1 siRNA (middle panel) and quantification of 53BP1 expression under the endogenous versus knock down condition across multiple cells (N>200 cells, error bar indicates SEM) (bottom panel) (Scale bars, 20 µm). **b.** Representative fluorescence intensity images of NB FFS data acquired of siRNA resistant eGFP-53BP1 before and 60 min after treatment with 4OHT in DIvA cells where endogenous 53BP1 has been knocked down (top row) and the corresponding brightness maps pseudo-coloured according to oligomeric state (middle row). Quantitation of this experiment across multiple cells (bottom row), including the fraction of siRNA resistant eGFP-53BP1 dimers in the nucleoplasm (left) as well as the fraction of nucleus occupied by siRNA resistant eGFP-53BP1foci (right), before and 60 min after 4OHT treatment (N = 5 cells, one biological replicate). Box and whisker plots show the minimum, maximum, sample median, and first versus third quartiles. (Scale bars, 2 µm).

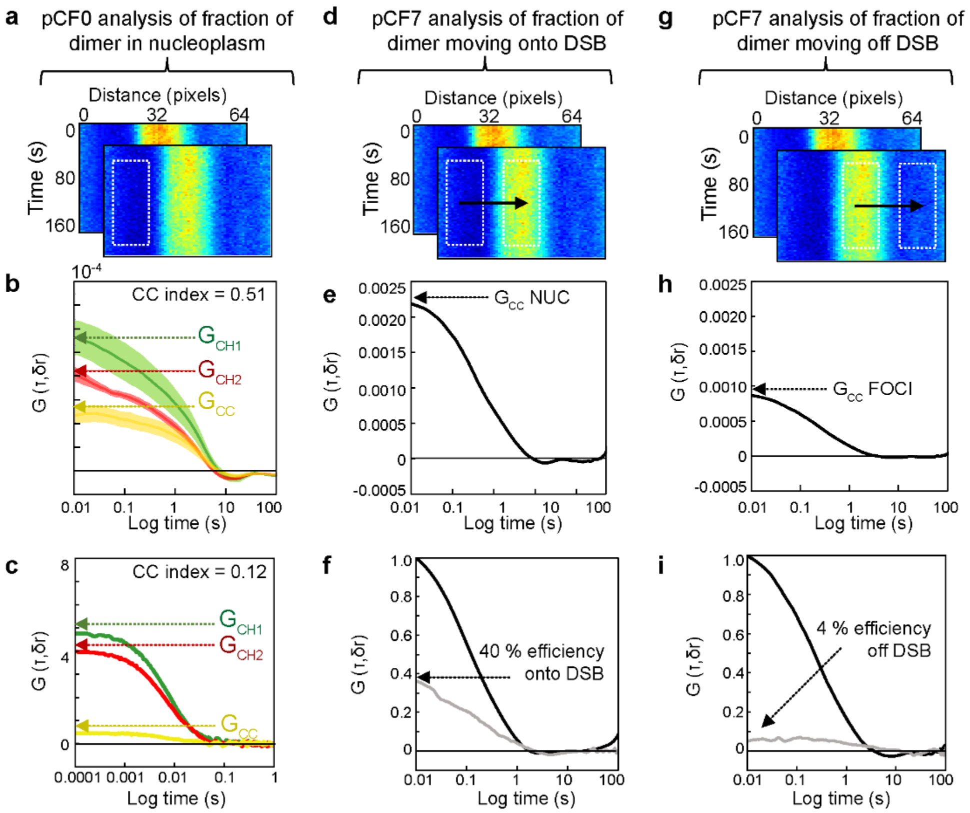

Supplementary Figure 5. Cross pair correlation function (pCF) analysis of the fraction of 53BP1 dimer present and the spatial evolution of 53BP1 dimer transport. a. To quantify the fraction of 53BP1 dimer present in the nucleoplasm along a two channel line scan we perform a pCF0 analysis (which is equivalent to the standard autocorrelation function) and compare it with a cross pCF0 analysis on pixels located in this region of interest (white dashed box). **b.** The fraction of 53BP1 dimer is equal to the ratio of the cross pCF0 amplitude at $\tau = 0$ ($G0_{CC}$) with the pCF0 amplitude of the limiting channel at $\tau = 0$ ($G0_{CH1}$ if $G0_{CH1} < G0_{CH2}$ or $G0_{CH2}$ if $G0_{CH2} < G0_{CH1}$) and this ratio is called the cross correlation index (CC index). **c.** A CC index for spectral bleed through was derived from simulation of a two-channel line scan acquired across a population of green molecules (CH1) exhibiting the experimentally measured percentage of eGFP signal detected in the mKate2 channel (CH2) in the presence of a population of red molecules (CH2). This resulted in cross pCF0 (yellow) and pCF0 profiles (green, red) that gave rise to a CC index of 0.12 and this bleed through CC index was subtracted from the CC index measured for 53BP1 dimerisation (b). **d-f.** To quantify 53BP1 dimer transport onto a DSB foci we perform cross pCF7 analysis on pixels located in the nucleoplasm (white dashed box) (d). This involves first determining the fraction of 53BP1 dimer present at the starting pixel positions via a cross pCF0 analysis (e) and then normalisation of the corresponding cross pCF7 profile with respect to the pCF0 amplitude (G_{CC}NUC) (f). **g-i.** To quantify 53BP1 dimer transport off a DSB foci we perform cross pCF7 analysis on pixels located in the foci (white dashed box) (g). This involves first determining the fraction of 53BP1 dimer present at the starting pixel positions via a cross pCF0 analysis (h) and then normalisation of the corresponding cross pCF7 profile with respect to the pCF0 amplitude (G_{CC}FOCI). The normalised cross pCF7 profiles presented in (f) and (i) have amplitudes that are indicative of the efficiency of these two different transits.

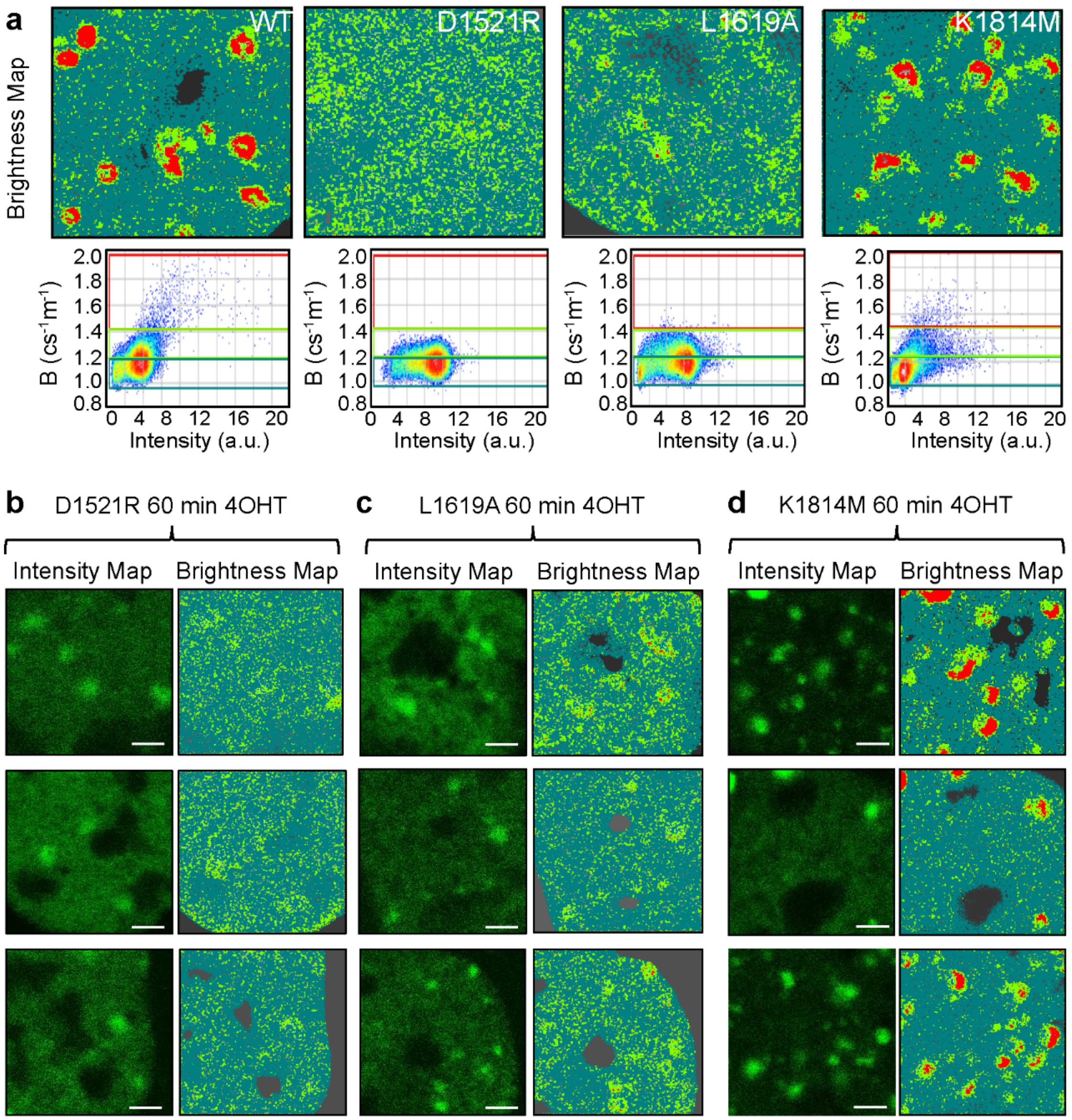

Supplementary Figure 6. Number and Brightness (NB) analysis of 53BP1 mutants that inhibit recognition of the DSB histone code in live DIvA cells. a. Brightness maps of the eGFP-53BP1 (WT), eGFP-53BP1^{D1521R} (D1521R), eGFP-53BP1^{L1619A} (L1619A), and eGFP-53BP1^{K1814M} (K1814M) NB experiments presented in Fig. 4f-h (top row) and the corresponding intensity vs brightness scatterplots (bottom row). **b-d.** Representative NB FFS data acquisitions from region of interests (ROIs) within different DIvA cell nuclei expressing D1521R (b), L1619A (c) and K1814M (d) 60 min after addition of 4OHT (c). (Scale bars, 2 μm).

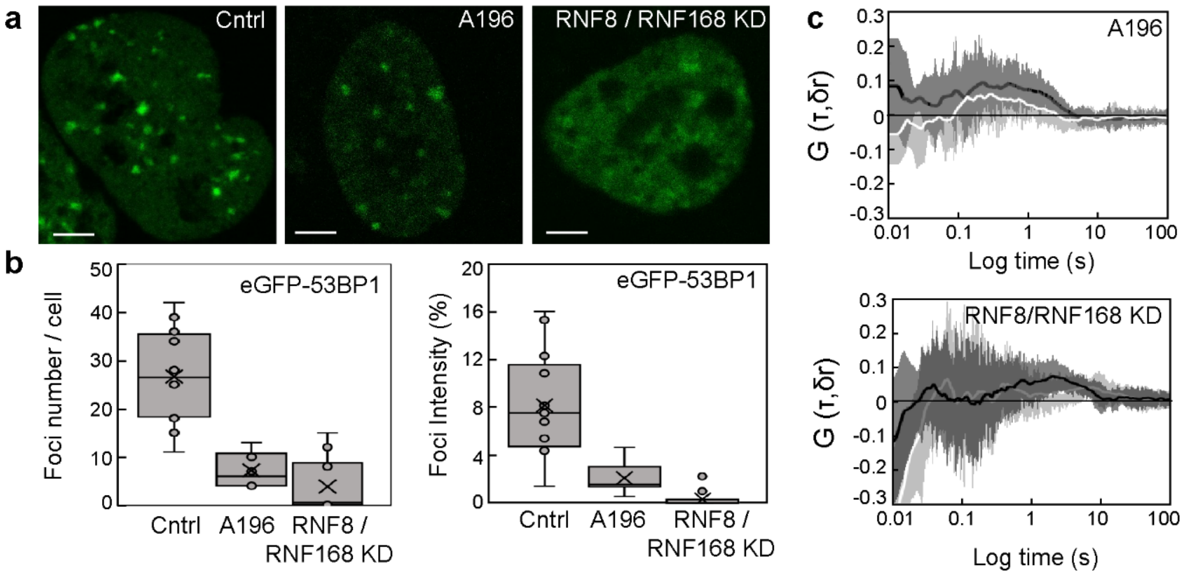

Supplementary Figure 7. Cross pair correlation function (pCF) analysis of 53BP1 dimer transport in the absence of RNF8/RN168 and SUV4-20h1/2. a. Representative fluorescence intensity images of eGFP-53BP1 in DIvA cells 60 min following 4OHT treatment in the absence versus presence of A196 treatment or RNF8/RNF168 double knock down. **b.** Quantification of eGFP-53BP1 foci number (left panel) versus foci intensity (right panel) in DIvA cells 60 min following 4OHT treatment in the absence versus presence of A196 treatment or RNF8/RNF168 double knock down. **c.** Cross pCF7 analysis of eGFP-53BP1 dimer translocation onto a DSB foci (black) versus off this nuclear structure (white) in DIvA cells treated with A196 (top panel, N = 10 cells) versus an RNF8/RNF168 double knock down (bottom panel, N = 7 cells) 60 min following 4OHT treatment. Cross pCF7 curves are normalised with respect to cross pCF0. Shading indicates SEM. All. Box and whisker plots show the minimum, maximum, sample median, and first versus third quartiles. (Scale bars, 5 μm).
